# Community perspectives on health professionals’ competence for quality primary health care in Amhara region, Ethiopia: a qualitative study

**DOI:** 10.1186/s12960-026-01068-w

**Published:** 2026-04-30

**Authors:** Gebeyehu Tsega, Getu Degu, Gizachew Yismaw, Mirkuzie Woldie

**Affiliations:** 1 Department of Health Systems Management and Health Economics, School of Public Health, College of Medicine and Health Sciences, Bahir Dar University, P.O. Box 79, Bahir Dar, Ethiopia; 2 Fenot Project, Department of Global Health and Population, Harvard T.H. Chan School of Public Health, Addis Ababa, Ethiopia; 3 Department of Epidemiology, School of Public Health, College of Medicine and Health Sciences, Bahir Dar University, P.O. Box 79, Bahir Dar, Ethiopia; 4 Health Research Development Directorate, Amhara Public Health Institute, Bahir Dar, Ethiopia; 5 Department of Health Policy and Management, Faculty of Public Health, Jimma University, Jimma, Ethiopia

**Keywords:** Primary health care, Health professional competence, Community perspectives, Quality of care, Universal health coverage, Ethiopia

## Abstract

**Background:**

Communities, as funders and users of the health system, have a vital role in ensuring the provision of integrated people-centered primary health care (PHC). However, there is a lack of information on community voices about the competence of health professionals in Ethiopia. Therefore, this study aimed to explore the community voices on the competence of health professionals for the provision of quality PHC services.

**Methods:**

A qualitative case study involving 10 key informant interviews and 6 FGDs was conducted from June 1 to July 30, 2023, in the Amhara Region among community representatives. The sample size was determined based on data saturation, and purposive heterogeneous sampling was employed. The high-quality health system framework and the Standards for Reporting Qualitative Research (SRQR) were used for analysis and report writing, respectively.

**Results:**

The key informants and discussants shared views regarding the competence of health professionals working in PHC facilities. These were categorized into three main themes and six subthemes. The main themes were quality impacts, process of care, and foundations. The subthemes were confidence in health professionals’ competence, health outcomes of the care provided by health professionals, economic benefits of the care, users’ experiences, competent care and system, and governance.

**Conclusions:**

The community had lost trust/confidence in the health professionals working at the PHC settings due to their negative experiences with them and forgone health care services. The community recommended that primary health care should have highly competent health professionals who receive adequate support, payment, working conditions, training, regulation, and who prioritize the people’s needs and expectations in their practices. This implies that the region has not yet achieved effective implementation of UHC principles through PHC. Thus, the regional and sub-regional governments and health professionals should work to rebuild the confidence (trust) of the community by paying attention to their concerns and pain points.

**Supplementary Information:**

The online version contains supplementary material available at 10.1186/s12960-026-01068-w.

## Introduction

Competent health professionals are fit-for-purpose professionals who possess the capability to deliver high-quality health care, ranging from prevention to palliative care services, utilizing the best available evidence, with the ultimate aim of enhancing the health and well-being of communities and patients [1]. According to the Global Competency Framework for UHC, health professionals should be equipped with six competency dimensions to provide quality primary health care. These dimensions include people-centeredness, communication, evidence-informed practice, decision-making, collaborative teamwork, and personal conduct/ethics [2]. Health professionals with these competencies are the backbone of quality primary health care (PHC), and without them, none of the PHC goals and targets will be achieved [3–7]. PHC is a whole-of-society approach for health and well-being with three key components: empowered people and community; multi-sectoral policy and action; and integrated health services (with prioritized delivery of high-quality primary care and essential public health functions) [8].

In low-income countries, including Ethiopia, health professionals’ indicators focus on the number, with less attention paid to assessing their competence from their own perspective and from the community’s view in the workplace, unlike in high-income countries [9, 10]. Although different health professional competence assessments such as multiple choice questions (MCQs) and objective structured clinical examinations (OSCEs), among others, have been conducted before and at the time of graduation (e.g., during exit exams) [11–14], information on the voices of the communities regarding the competence of health professionals in their workplace is missing.

Recently, there has been a growing call for high-quality health systems that provide integrated people-centered PHC to ensure people’s right to health [11, 15]. As funders and users of the health system, the voices of communities on health professionals’ competence really matter [7, 9, 10]. Their voices reflect the extent to which health professionals satisfy and respond to people’s needs, such as in providing care with dignity and respect (people-centered care) and people’s trust (confidence) in health providers [11]. These voices are very important for the health systems to be people centered and can provide valuable evidence to policy/decision-makers and health professionals. However, this evidence is not available in Ethiopia in general, nor in the Amhara region in particular. Hence, in this study, we set out to address this gap by collecting qualitative data on the community’s voices regarding health professionals’ competence to provide quality PHC, notably PHC settings, which are the only, if not the main, health care service providers in the rural settings of the Amhara region, Ethiopia. Such evidence is especially relevant to the Amhara region—one of Ethiopia’s conflict-affected areas—as it supports developing context-specific interventions to strengthen the resilience and responsiveness of the PHC system. By addressing both current and changing health needs caused by conflict, the findings offer strategic guidance for improving the adaptive capacity of health professionals and PHC to maintain, restore, and expand essential services during crises.

## Methods

### Study design and research paradigm

Between June 1 and July 30, 2023, a qualitative case study was conducted. The constructivist philosophical assumption served as the foundation. This philosophical stance is closely associated with qualitative methods and informal rhetoric. Consequently, the researchers opted for a qualitative case study. This approach involves collecting data from various sources and considering diverse perspectives [16]. Researchers place significant emphasis on participants’ viewpoints and subjectively interpret the observed phenomena. Constructivist research unfolds from individual perspectives, gradually revealing broader patterns and ultimately leading to comprehensive understandings [17].

### Researcher characteristics and reflexivity

All the researchers have a background in health sciences and possess substantial knowledge and experience in both qualitative and quantitative research. All of them believe in a pragmatic philosophical foundation [18].

### Study setting

The study was conducted in the Amhara Region, with a special emphasis on the PHC setting. Recognizing that PHC serves as the cornerstone for achieving Universal Health Coverage (UHC) and provides 90% of essential services [19], we specifically focused on the PHC setting within the Amhara Region. The Amhara Region is one of the most populous regional states in Ethiopia and is also an area affected by armed conflict. It comprises 14 zones, 8 city administrations, and 266 districts [20]. The region has a total of 32,589 health professionals [21].

### Study participants

Selected community representatives from three zones and three town administrations—including Client Council members of the community score card, village health leaders, and kebele leaders within the Amhara region’s PHC facilities—served as study participants. The study participants were selected purposively and included heterogeneous groups of individuals to get diverse insights on health professionals’ competence. Therefore, ten key informant interviews (KIIs) with individuals knowledgeable about relevant information (such as kebele leaders) and six focus group discussions (FGDs) with 6–7 village health leaders or client councils in each group, with a total of 40 participants, were conducted until information saturation was reached [22, 23]. Members of client councils—community representatives responsible for ensuring the social accountability of health facilities—and village health leaders were included in the FGDs because they are well‑informed about community concerns. For example, each village health leader is expected to provide community health services for approximately 75–100 households, a role introduced in 2020 in Ethiopia, including the Amhara region. Information saturation represents a point of information redundancy or diminishing returns, indicating the juncture at which sufficient information power is attained [24].

### Data collection

The data were collected (by three master holder data collectors and the first author) through KIIs and FGDs with mean durations of 50 min and 80 min, respectively. Both KIIs and FGDs were conducted in the local language (Amharic). Probing was performed to reveal further details when the study participants mentioned points of significance with limited details. The interview guides were prepared (in English) based on previous literature [2] and comprise key and sub-questions. Then the English version was translated into the local language (Amharic) by the translator experts. To prioritize participants’ comfort and privacy during interviews and focus group discussions (FGDs), we carefully selected appropriate settings. For key informant interviews, we opted for quiet rooms within health facilities or kebeles’ offices. Moreover, closed meeting rooms or community halls were used for FGDs. Our primary goal was to establish a supportive environment that encourages open communication, avoids sound disturbance during recordings, and acknowledges each participant’s need for privacy and comfort. Audio recordings and field notes were kept during data collection.

### Trustworthiness

The trustworthiness of data was assured as per Guba and Lincoln’s trustworthiness criteria [25]. Trustworthiness of the data was ensured through multiple strategies: credibility was established by prolonged engagement, triangulation, and member checking; transferability was supported by thick description and heterogeneous purposive sampling; dependability was maintained using a codebook, audit trail, peer debriefing, and negative case analysis; and confirmability was strengthened through reflexivity, audit trail, and peer debriefing. In addition, audio‑taped data were transcribed, translated, and continuously cross‑checked against recordings to guarantee accuracy.

### Data management and analysis

Data collection and analysis were conducted simultaneously. Verbatim transcription and conceptual translation were conducted by the first author. Codes were first generated through content analysis and then examined using thematic analysis with ATLAS.ti 9 to identify patterns across participant narratives. To deepen interpretation, we subsequently applied framework analysis—guided by the High‑Quality Health System Framework—to organize the themes into predefined domains: foundations, processes of care, and quality impacts [11]. Moreover, the Standards for Reporting Qualitative Research (SRQR) informed the process and guided the reporting of findings. These standards enhance the transparency of all aspects of qualitative research by providing clear guidelines for reporting [26].

## Results

### Background characteristics of study participants

The study involved 10 key informants and 40 discussants across six focus groups. Key informants ranged in age from 20 to 60, with educational backgrounds from no formal education to degree holders, and occupations including farming and public sector jobs. Discussants were aged 23 to 50, with education levels from no formal education to a diploma, and most were farmers (Additional file 1).

### Community’s voices on health professionals’ competence

The key informants and discussants shared their views regarding the competence of health professionals. These were categorized into three main themes and six subthemes. The main themes were quality impacts, process of care, and foundations. The subthemes were confidence in health professionals’ competence, health outcomes of the care provided by health professionals, economic benefits of the care, users’ experiences, competent care and system, and governance.

### Quality impacts

Key informants and discussants highlighted a range of complex issues, including poor health outcomes, low confidence in the health professionals, and financial hardship linked to perceived incompetence in primary health care facilities. This theme was organized into three subthemes: health outcomes, confidence, and financial hardship.

### Health outcomes

Most respondents reported that some health professionals failed to cure the diseases and sometimes harmed them, with limited public health services. As a result, unsafe care and medical errors made by health professionals led to morbidities, disabilities, and deaths. For example, one discussant reported:

… my child was sick and went to a health center for health services. However, they [health professionals] provided treatment that was not aligned with his health problem, and as a result, he was not cured there…This situation should be improved (Male, 50 years old, FGD).

Essential public health functions (EPHFs) play a crucial role within the integrated health services dimension of PHC. Nearly all respondents reported that health professionals working in PHC units tend to overlook essential public health functions. Instead, they delegate EPHFs to health extension workers who are insufficiently trained for these tasks as perceived by the people. This results in low health literacy among the community members, which undermines people’s and community empowerment and the integrated health PHC service delivery.

### Confidence

Most respondents reported that community members have lost confidence in health professionals because of the negative experiences they encounter. They reported that the health professionals at PHC units treated the community and patients as inferior, and many health professionals adopted a paternalistic approach, making decisions without considering the needs, preferences, and values of the community and patients. For example, a discussant complained:

… The same health professionals behave differently in their private clinics and in public health facilities…The people live in between light and dark (Female, 48 years old, FGD).

### Financial hardship

Most respondents reported that the community (people) faced economic burdens with catastrophic health care expenditures leading to impoverishment. This is due to medical errors, delayed provision of services, (unnecessary and delayed) referrals, and costs to access care from the secondary and tertiary levels or a private clinic. For example, a respondent reported:

Most people, unless they are very poor, prefer the private health sectors… However, the cost is extremely high. For very poor people, the only option for receiving health services is public health centers and primary hospitals… Therefore, to get timely care, you have to be either a relative or a friend of the health professionals there, or you have to pay a lot of money for health services in the private clinics (Female, 43 years old, KII).

### Process of care

As highlighted by all respondents, many undesirable health outcomes stem from inadequate care processes, which fail to establish a solid foundation for quality health care. This theme is organized and described through two subthemes: users’ experiences and competent care and the system.

### Users’ experiences

All respondents reported that the community (people) often experiences long waiting and appointment times and short consultation periods. For example, a respondent reported:

To get a consultation from a specialist, you may have to wait up to a month, while the specialists of the hospitals are available at any time at their private clinics… (Female, 38 years old, FGD).

### Competent care and system

All respondents, except one, reported that the care process quality was poor. The respondents reported critical issues such as failure to explain the diagnosis, not advising on what to do and what to avoid, and sometimes abusive behavior among the health professionals. They also complained about the very limited resources due to the artificial shortage of drugs, and the lack of dignity and respect in the provision of care. The respondents pointed out that some health professionals favor their relatives, networks, friends, and non-community-based health insurance users. Due to these problems, people have forgone the health services of PHC units.

For example, a respondent explained, “…* some health professionals are not competent and may even cause harm to patients. As a result, people in our kebele have forgone health services provided by the health center and instead use traditional health care methods*… (*Male, 35 years old, FGD*).

### Foundations/governance

Most respondents reported that health professionals working in health centers and primary hospitals are not well-regulated, supported, or paid. Some health professionals can get their salary even if they are absent from their workplace. Few health professionals can fraud drugs and either sell them or use them in their own clinics. They are not held accountable or responsible for their actions and decisions. There is no functional system to ensure their competence and ethics. There is no culture of quality, evidence-informed management, supervision, and feedback in these health facilities. The health professionals only provide service in health facilities with very limited community-based health programs (e.g., irregular outreach programs for immunization). The government does not address the health professionals’ issues, such as duty payments and basic needs. This is reflected by respondents’ reports that services were interrupted several times due to strikes by health professionals since their duty payments and benefit packages were not provided. For example, a respondent reported:

When we give feedback to some health professionals to improve their service, they tell us that, if necessary, they can change their workplace and that their salary will not be affected… some health professionals working here are rude and have bad attitudes. For example, one health professional who has been informed [by the community] three times to improve his rude behavior, such as ethical misconduct, threatened the patients by saying ‘You cannot do anything, I can change my workplace (Male, 49 years old, FGD).

Many respondents also complained that some health professionals are more focused on making money than on serving people, even though they are supposed to provide people-centered care. They said that those health professionals are not punished for their unethical practices, which encourages them to continue or worsen their behavior in the future. They also said that they had reported these problems to the local authorities through various channels, including community score card meetings. For example*,* a discussant reported:

Few health professionals have turned the health center into a business hub for private clinics and pharmacies; we noted that most of the private pharmacies and clinics near the public health facilities are owned by the health professionals who work there… (Male, 41 years old, FGD).

Table 1 summarizes the themes and subthemes with key issues and illustrative quotes. It provides a concise synthesis of the findings and highlights the concerns most frequently raised by community members.

**Table 1:** Themes and subthemes on community perspectives of health professionals’ competence for primary health care, Amhara region, Ethiopia, 2024

Themes	Subthemes	Key issues	Quotes
Quality impacts	Health outcomes	Unsafe care, medical errors, poor disease management, neglect of essential public health functions	“My child was sick… they provided treatment not aligned with his health problem…” (Male, 50 years old, FGD)
	Confidence	Loss of trust/confidence, paternalistic attitudes, disrespect, inconsistent behavior in public and private sectors	“…The same health professionals behave differently in their private clinics…” (Female, 48 years old, FGD)
	Financial hardship	Catastrophic expenditures, delayed referrals, reliance on costly private care, impoverishment	“Most people… prefer the private health sectors… the cost is extremely high.” (Female, 43 years old, KII)
Process of care	Users’ experiences	Long waiting and appointment times and short consultations period	“To get a consultation… you may have to wait up to a month…” (Female, 38 years old, FGD)
	Competent care and system	Poor communication, abusive behavior, unfair care, artificial drug shortages, lack of dignity	“… some health professionals are not competent and may even cause harm…” (Male, 35 years old, FGD)
Foundations	Governance	Weak regulation, absenteeism, fraud, unethical practices, lack of supervision, strikes due to poor incentives	“Few health professionals have turned the health center into a business hub…” (Male, 41years old, FGD)

## Discussion

The aim of this study was to explore the voices of the community on health professionals’ competence for the provision of quality PHC in the Amhara region, Ethiopia. This study revealed that the community expressed their concerns about what is going on in primary health care facilities in the region. Health professionals are not only blamed for technical incompetence but also for unethical behavior rampantly observed in the facilities. These concerns were raised in all three themes: the impact of care, the care process, and the foundations supporting the health care delivery.

This study found that the community’s perceptions of PHC provision were not in line with the expected impacts of PHC [8, 11, 27, 28], such as improved health and well-being, trust, and economic benefits to the community. Community members expressed dissatisfaction with health professionals’ competence due to receiving low-quality, unsafe, and error-prone PHC. They also struggled with financial hardship due to the high costs of seeking better care from higher-level facilities or private clinics. This mainly relates to the fact that PHC units have poor working conditions, which make it challenging to attract and retain competent health professionals. These include low pay, no incentives for serving in remote or risky areas, insecurity, lack of infrastructure (such as water, electricity, housing, roads, and networks), limited training opportunities, insufficient health system support, unfair and ineffective management of the health workforce, and lack of recognition and regulation [9–11, 29]. This result is supported by the findings of the service provision assessment conducted in 2021/2022, which showed that fewer than one-fifth (18%) of health care professionals correctly diagnosed malaria with anemia [30]. This implies that the community members perceived that health professionals were not equipped with the required competencies to provide quality primary health care. Consequently, the care provided may not lead to desirable health outcomes due to non-adherence to their advice and prescribed drugs, even though health professionals can provide good care for the clients. As a result, people may choose not to use or fund the primary health care because they perceive that health professionals cannot meet their health care needs there. Instead, they prefer to bypass lower-level health facilities and seek care at higher-level or private health facilities, which are often more expensive. Trust is fundamental for the provision of health care. Without trust, providing good care that satisfies the needs of clients is almost impossible [31, 32]. As the findings revealed, achieving Universal Health Coverage (UHC), which includes quality essential health services access and financial protection, can be challenging in the region due to the current state of primary health facilities and the perceived low competence of health professionals. However, Ethiopia, in general, and the Amhara region, in particular, prioritize primary health care as a key focus and main approach for achieving health-related sustainable development goals, including UHC, as reflected in the revised health policy [33] and the current health sector strategic plan called the health sector medium-term development and investment plan (HSIP) [34].

The current study also revealed that the process of PHC services falls short of its promise of competent care/system and positive user experiences [11]. The community members reported that they often faced negative experiences such as disrespectful and abusive care, long waits and appointment times, short consultation periods, and incompetent care (by less competent providers without clear explanations) and system (with errors and bias) in PHC. The respondents reported that the community members did not participate in the decision-making of their care. This finding is supported by a recent study, which stated that only 39% of clients in Ethiopia were actively involved in decision-making during the receipt of health care from health providers [35]. This is because health professionals lack adequate support, training (pre- and in-service), regulation, pay, and motivation in PHC and beyond [36–38]. This implies that health professionals require comprehensive support, training, and regulations to enable them to provide people-centered primary health care. Without people-centered quality PHC, the region will not achieve its health priorities.

According to the high-quality health system framework, the regulations and governance of health professionals are fundamental for positive health outcomes, as they consider the needs and expectations of the population, the organizational culture, and other factors [11]. However, the community members reported that the primary health care (PHC) settings had weak and ineffective health regulations and legal frameworks, a lack of quality improvement, a lack of collaborative practice and a continuous learning organizational culture, and a fragmented organization of care. The organization of care was mainly based on a biomedical model that was limited to health facilities with episodic encounters, rather than community-based and continuous care. This means that the Amhara region did not keep its promise in achieving universal health coverage (UHC) through primary health care (PHC) and health policies were not translated into actions. This is mainly due to the fact that the region has faced numerous challenges that exacerbated existing health problems. These challenges include conflict, disease outbreaks like COVID-19, and climate change effects such as drought and food insecurity. According to the United Nations Secretariat, conflict creates health crises that impact the well-being and security of health professionals, subsequently affecting their competence [29]. While the trustworthiness of the findings was ensured using Guba and Lincoln’s criteria—credibility, transferability, dependability, and confirmability—the results represent community members’ perceptions, which may not fully correspond to the actual competence of health professionals in delivering quality primary health care. These perspectives offer valuable insights into perceived responsiveness, respect, and trust; however, they do not constitute objective evaluations of health professionals’ competence.

## Conclusions

The community expressed dissatisfaction with the competence of health professionals, who did not meet their needs and expectations. The community had lost trust in health professionals’ competence for the PHC due to negative experiences with them. This resulted in poor health outcomes, financial hardship, and low confidence in health professionals. In summary, this study showed that the empowered community and people dimension of PHC were overlooked in the region.

## Implications for policy and practice

To address the community’s concerns and expectations, we recommend the following actions:

- Improving health professionals’ competence by providing continuous professional development, supervision, feedback, standards, and guidelines.
- Restoring the community’s confidence in health professionals by increasing community engagement and promoting social accountability through tools like the community score card.
- Optimizing the use of available resources and seeking additional sources of funding for PHC services.
- Strengthening evidence-informed decision-making on the health professionals’ competence.

## Supplementary Information

Additional file 1.

## Data Availability

The datasets used and/or analyzed during the current study are available from the corresponding author on reasonable request.

## Abbreviations

COVID-19: Coronavirus disease of 2019
CSC: Community Score Card
MoH: Ministry of Health
PHC: Primary Health Care
UHC: Universal Health Coverage
WHO: World Health Organization
