# Supplementary material for: Community perspectives on health professionals’ competence for quality primary health care in Amhara region, Ethiopia: a qualitative study
Source: Hum Resour Health. 2026 Apr 30;24:32. doi: 10.1186/s12960-026-01068-w (PMC13312639; doi:10.1186/s12960-026-01068-w)
Supplement: Supplementary file 1 — Additional file 1. [file 12960_2026_1068_MOESM1_ESM.docx]

Additional file 1: Background characteristics of key informant interviewees and discussants, Ethiopia, Amhara region, 2024

| Background characteristics of key informant interviewees | | | | | | | |
| --- | --- | --- | --- | --- | --- | --- | --- |
| Code | Age | Education | Sex | Code | Age | Education | Sex |
| KII 1 | 40 | Degree | Male | KII 6 | 43 | degree | Female |
| KII 2 | 20 | Formally uneducated | Female | KII 7 | 35 | 10^th^ grade | Male |
| KII 3 | 35 | Formally uneducated | Female | KII 8 | 34 | 12^th^ grade | Female |
| KII 4 | 45 | Diploma | Male | KII 9 | 33 | 8^th^ grade | Male |
| KII 5 | 60 | Formally uneducated | Male | KII 10 | 42 | Diploma | Male |
| Background characteristics of discussants | | | | | | | |
| Code | Age | Education | Sex | Code | Age | Education | Sex |
| FGD 1 | 41 | 8th grade | Female | FGD 21 | 30 | Formally uneducated | Female |
| FGD 2 | 30 | Diploma | male | FGD 22 | 31 | Formally Uneducated | Male |
| FGD 3 | 36 | Diploma | Male | FGD 23 | 50 | Diploma | Male |
| FGD 4 | 25 | 8th grade | Male | FGD 24 | 37 | Diploma | Male |
| FGD 5 | 48 | 10th grade | Female | FGD 25 | 25 | Formally uneducated | Female |
| FGD 6 | 25 | 8th grade | Female | FGD 26 | 33 | Diploma | Male |
| FGD 7 | 35 | 4th grade | Male | FGD 27 | 28 | 8th grade | Female |
| FGD 8 | 27 | diploma | Female | FGD 28 | 32 | Diploma | Female |
| FGD 9 | 37 | 6th grade | Male | FGD 29 | 33 | Diploma | Male |
| FGD 10 | 30 | 10th | Male | FGD 30 | 30 | 12th grade | Male |
| FGD 11 | 38 | Degree | Female | FGD 31 | 30 | 6th grade | Female |
| FGD 12 | 23 | Formally uneducated | Female | FGD 32 | 30 | Diploma | Female |
| FGD 13 | 30 | 8th grade | Male | FGD 33 | 40 | Diploma | Male |
| FGD 14 | 31 | Diploma | Male | FGD 34 | 30 | 8th grade | Male |
| FGD 15 | 35 | Diploma | Female | FGD 35 | 35 | First degree | Female |
| FGD 16 | 29 | 8th grade | Male | FGD 36 | 28 | First degree | Male |
| FGD 17 | 28 | 10th grade | Male | FGD 37 | 25 | 8th grade | Male |
| FGD 18 | 27 | 8th grade | Male | FGD 38 | 30 | 10th grade | Male |
| FGD 19 | 49 | Diploma | Male | FGD 39 | 24 | 8th grade | Male |
| FGD 20 | 30 | Diploma | Male | FGD 40 | 29 | Diploma | Male |
